# Wzc mutation enhances virulence in CRKP ST11-KL64 via complement-mediated lysis and lipopolysaccharide release

**DOI:** 10.1128/spectrum.02847-25

**Published:** 2026-02-27

**Authors:** Huanhuan Yan, Yufeng Dai, Kun Ye, Qiang Zhao, Na Guo, Lifeng Wang, Jiyong Yang

**Affiliations:** 1 Department of Laboratory Medicine, The First Medical Center of Chinese PLA General Hospital 645732, Beijing, China; 2 Medical School of Chinese PLA 104607 , Beijing, China; Second Affiliated Hospital of Soochow University, Suzhou, China

**Keywords:** Carbapenem-resistant *Klebsiella pneumoniae*, ST11-KL64, Wzc, capsular polysaccharide, lipopolysaccharide

## Abstract

Carbapenem-resistant *Klebsiella pneumoniae* (CRKP) ST11-KL64 has emerged as the dominant subclone in China. Mutations in Wzc confer a hypermucoviscous (HMV) phenotype and enhanced virulence, but how these mutations regulate capsular polysaccharide (CPS) architecture, whether they alter virulence traits, and the mechanisms and consequences of these alterations remain unclear. We found that the Wzc_Y572D_ mutation in CRKP ST11-KL64 induced HMV phenotype, increased cell-free CPS production, and elongated CPS chain length. In a mouse peritonitis-septicemia model, Wzc mutant strains caused higher lethality, greater cytokine release, more pronounced inflammatory cell infiltration, and severe tissue damage. These mutants also exhibited increased anti-phagocytic ability and greater serum killing activity compared to the non-mutant strain. Elevated lipopolysaccharide (LPS) levels were detected in serum from mice infected with the mutant strains and in normal human serum incubated with these strains. The mutants showed stronger binding to C5b-9 than non-mutation strains. Notably, pretreatment of mice with TAK-242 reduced the lethality induced by CRB113Wzc_Y572D_. Collectively, these findings indicate that Wzc mutation in CRKP ST11-KL64 alters CPS spatial distribution and chain length, leading to HMV phenotype acquisition. These changes enhance complement-mediated bacterial lysis. The lysis of bacterial cells results in increased LPS release, and subsequent stronger inflammatory response and more severe tissue damage, ultimately enhances bacterial virulence.

**IMPORTANCE:**

Carbapenem-resistant *Klebsiella pneumoniae* (CRKP) ST11-KL64 combines multidrug resistance with elevated virulence, posing a significant public health threat. This study demonstrates that Wzc mutation modulates bacteria-host interactions and enhances bacterial pathogenicity through regulating capsular polysaccharide (CPS) architecture. Our findings provide novel insights into the interplay between CPS biosynthesis, the hypermucoviscous phenotype, and virulence in CRKP ST11-KL64.

## INTRODUCTION

Carbapenem-resistant *Klebsiella pneumoniae* (CRKP) has emerged as a major public health threat due to multidrug resistance (1, 2). In China, sequence type (ST) 11 is the most prevalent clone. A subclone shift in CRKP ST11 was observed: the previously dominant capsular locus (KL) 47 was replaced by KL64 (3–6).

The hypermucoviscous (HMV) phenotype is a critical hallmark of hypervirulent *Klebsiella pneumoniae* (hvKP) (7). HMV strains can evade phagocytosis and complement-mediated killing, promoting invasion and dissemination (8). Current studies report varied drivers of the HMV phenotype, including capsular polysaccharide (CPS) production, chain length, cell-associated CPS, or cell-free CPS (9–12). CPS synthesis is encoded by the chromosomal *cps* gene and regulated by multiple genetic factors, including the plasmid-borne *rmpADC/A2D2* locus (13, 14). The *rmp* locus contributes to the HMV phenotype and virulence (11, 15). However, CRKP strains exhibit the HMV phenotype without these known determinants, and acquisition of the virulence plasmids does not always result in a thick CPS but still enhances virulence, suggesting that other factors contribute to the HMV phenotype and virulence (16, 17). *wzc* within the *cps* gene cluster encodes an inner membrane tyrosine kinase essential for the polymerization and export of CPS chain via cycles of monomer phosphorylated and octamer dephosphorylation (18). Single-nucleotide polymorphisms (SNPs) in *wzc* have been shown to influence the HMV phenotype and virulence (12, 19–21). Wzc variants can enhance bacterial virulence by evading phagocytosis and host cell adhesion (19–21). Notably, unlike *rmp*-mediated mucoid phenotypes, Wzc-induced HMV phenotype exhibits lower serum resistance (8, 21, 22). This raises questions about their overall impact on virulence.

Understanding how *wzc* SNPs influence HMV phenotype and virulence is critical for elucidating CRKP pathogenicity. Here, we investigated the role of the Wzc mutation in the HMV phenotype, CPS architecture, and pathogenicity of CRKP ST11-KL64 both *in vitro* and *in vivo*. We found that the Wzc mutation promotes the HMV phenotype by altering CPS spatial distribution and chain length. Meantime, the HMV phenotype subsequently made the bacteria increasingly sensitive to complement-mediated lysis and elevating lipopolysaccharide (LPS) release, thereby enhancing CRKP ST11-KL64 virulence.

## RESULTS

### Wzc mutations impact mucoviscosity

**Fig 1:**
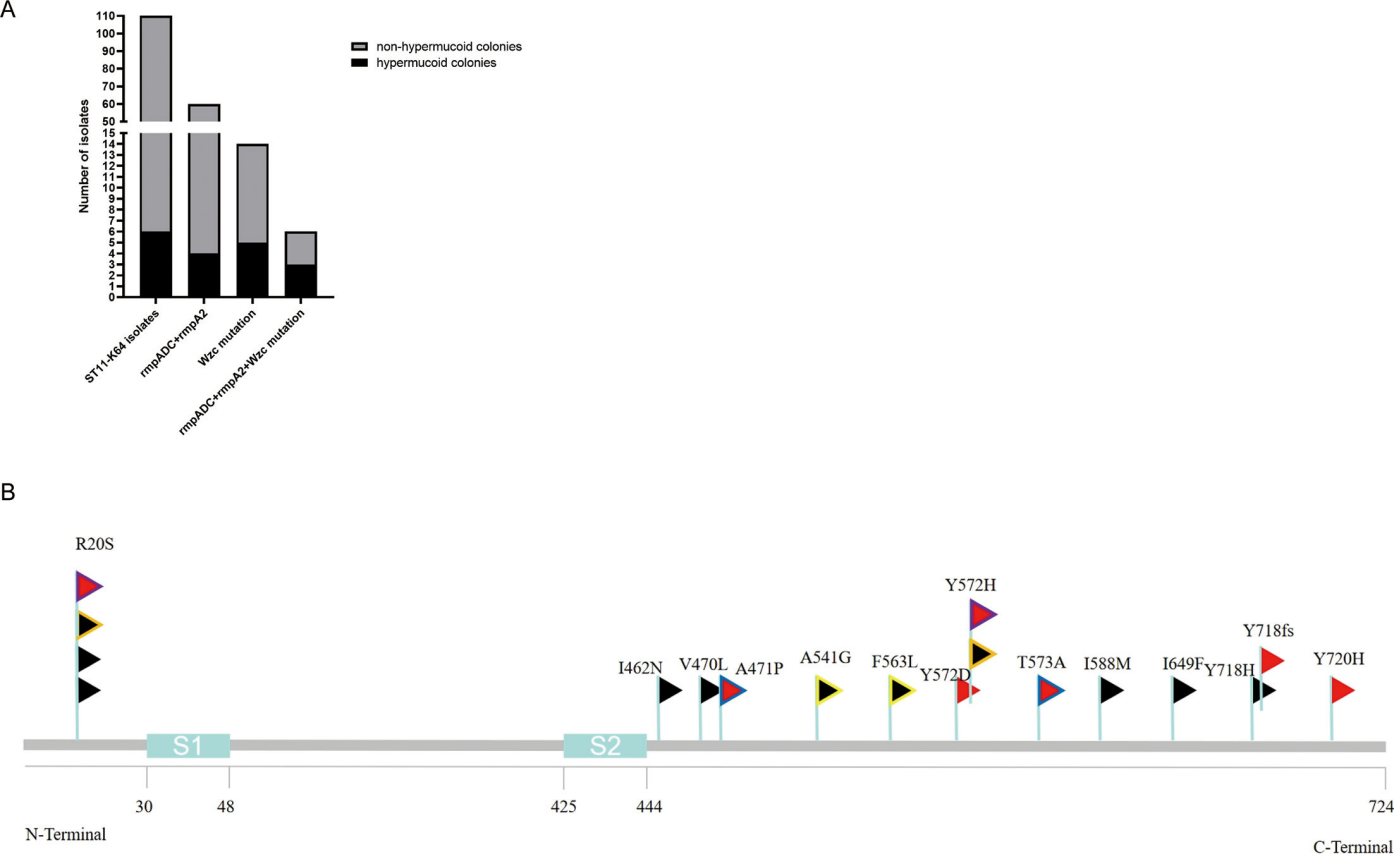
(**A**) Frequency of mucoid phenotypes of strains isolated. (**B**) Non-synonymous mutations found in Wzc in the screened collection of ST11-K64 isolates. The position of the mutations was indicated. Flags sharing some border color were indicative of multiple mutations found in an isolate. The mucoid phenotypes were indicated by the color of the flag: red, hypermucoid colonies; black, non-hypermucoid colonies. The predicted transmembrane segments (S1–S2) of protein were indicated.

We retrospectively analyzed 299 bloodstream infection (BSI) CRKP isolates, among which ST11-KL64 was the most prevalent, accounting for 110 isolates. All 95 ST11-KL64 isolates carried at least 3 virulence genes. Six of these isolates exhibited a hypermucoid colony phenotype (Fig 1; Table S1). Among 60 isolates co-harboring RmpADC and a frameshift-mutated RmpA2, 4 displayed the hypermucoid colonies (Fig. 1; Table S1). Notably, hypermucoid colonies were observed in 5 of 14 isolates that carried Wzc mutations, including 3 strains harboring Wzc mutations along with RmpADC and a frameshift-mutated RmpA2 (Fig. 1; Table S2). The most frequent Wzc mutation occurred at position 20, followed by a mutation one at position 572 (Fig. 1; Table S2). These findings suggest that Wzc mutations contribute to hypermucoid phenotype in CRKP ST11-KL64.

**Fig 2:**
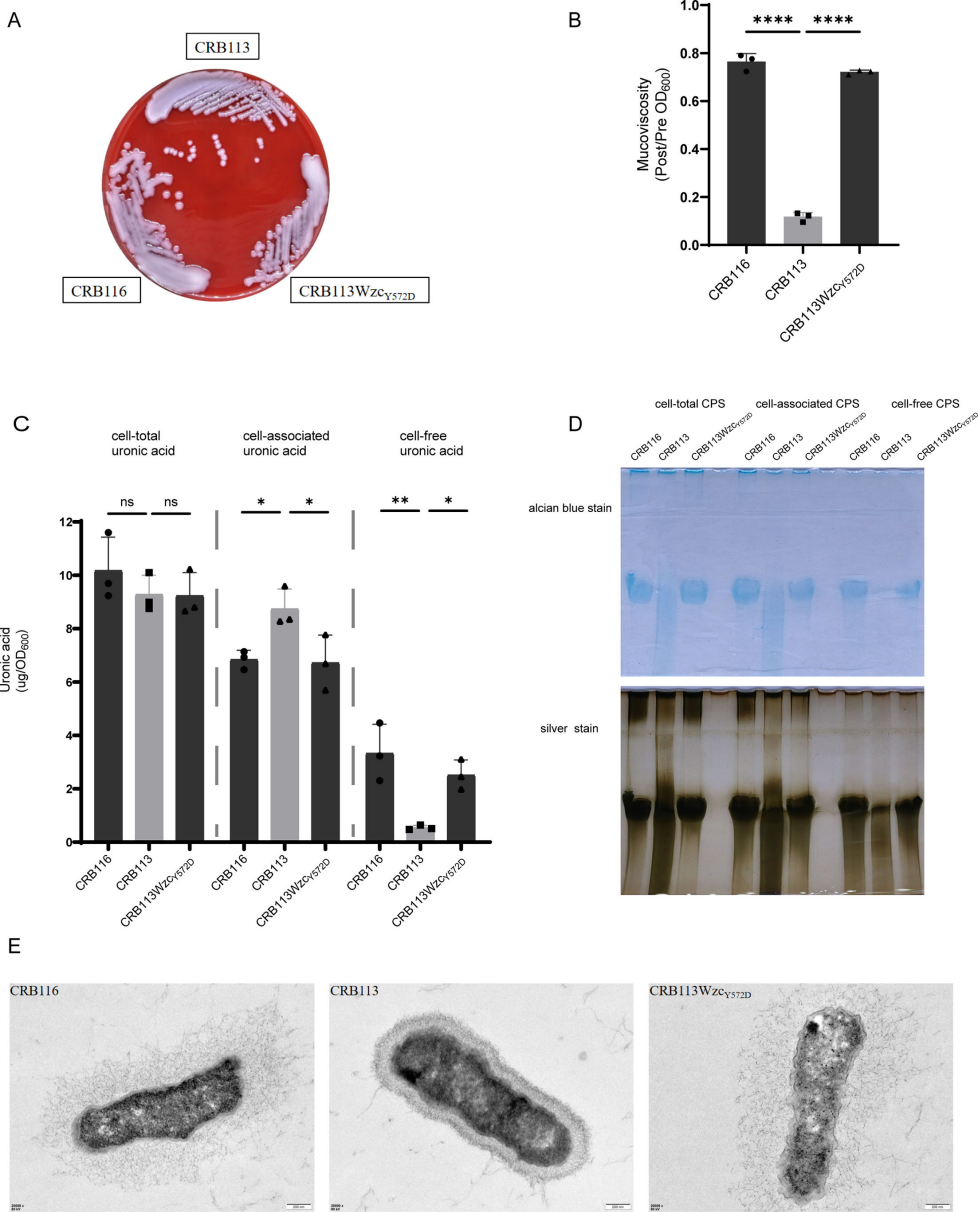
Hypermucoviscous (HMV)-related characteristics of CRB116, CRB113, and CRB113Wzc_Y572D_. (**A**) Colony morphology of the strains cultured on blood agar plates. (**B**) Sedimentation assay for mucoviscosity quantification. HMV phenotype ≥ 0.2, non-mucoviscous phenotype < 0.2. *n* = 3 biological replicates, bars indicate mean ± SD. ****P < 0.0001. (**C**) Uronic acid quantification. *n* = 3 biological replicates; bars indicate mean ± SD. ns *P* ≥ 0.05, *P < 0.05, and **P < 0.01. (**D**) Alcian blue staining (up) and silver staining (down) of capsular polysaccharide resolved on SDS-PAGE. (**E**) Transmission electron microscopy.

### Wzc_Y572D_-induced HMV phenotype correlates with CPS spatial distribution and chain length

To assess the impact of the Wzc mutation on the mucoid phenotype, we compared the HMV strain CRB116 (Wzc_Y572D_) with its phylogenetically related non-mucoviscous (NMV) strain CRB113 (Wzc wide type). Both strains carried RmpADC and a frameshift-mutated RmpA2. The Wzc_Y572D_ mutation was introduced into CRB113 using CRISPR/Cas9 genome editing, generating the engineered strain CRB113Wzc_Y572D_. This strain displayed an HMV phenotype and formed hypermucoid colonies (Fig. 2). The result indicated that the Y572D substitution in Wzc is sufficient to confer the HMV phenotype.

We next quantified CPS production and analyzed CPS chain length to determine how the Wzc mutation affects CPS formation. Cell-total uronic acid levels did not differ significantly among the strains (Fig. 2). In contrast, the HMV strains CRB116 and CRB113Wzc_Y572D_ exhibited reduced cell-associated uronic acid and concomitantly increased cell-free uronic acid compared with the NMV strain CRB113 (Fig. 2). CPS chain length analysis showed that the HMV strains CRB116 and CRB113Wzc_Y572D_ produced more uniform CPS chains, whereas the NMV strain CRB113 generated a broader distribution of CPS chain lengths (Fig. 2). Electron microscopy further revealed distinct CPS spatial organization: the HMV strains CRB116 and CRB113Wzc_Y572D_ displayed a bulky but disordered and sparse CPS layer, in contrast to the compact and uniform CPS layer observed in the NMV strain CRB113 (Fig. 2). Together, these results demonstrate that the Y572D substitution in Wzc alters CPS spatial distribution and chain length, thereby driving the HMV phenotype.

### Wzc_Y572D_ impacts bacterial virulence

**Fig 3:**
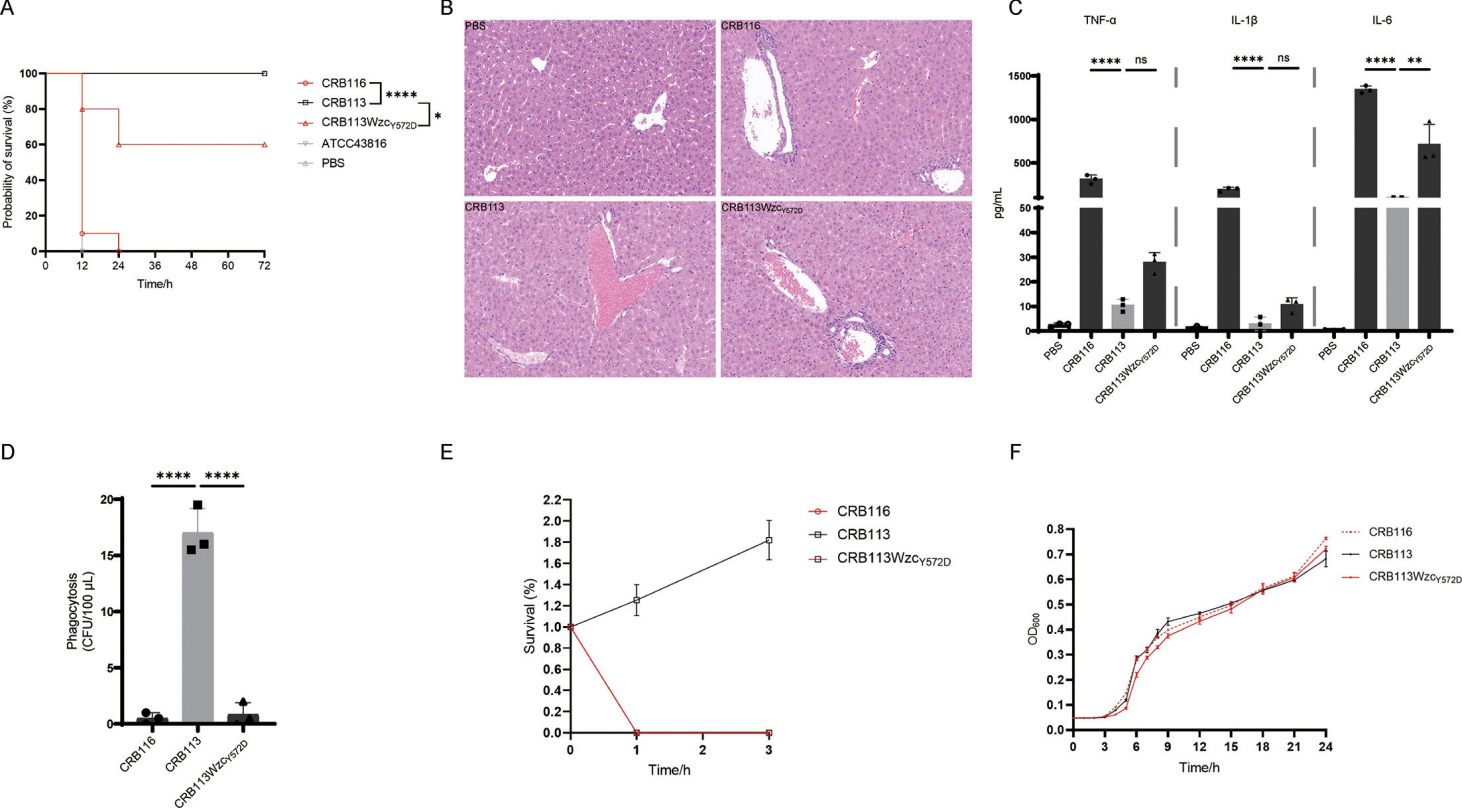
Virulence-related characteristics of CRB116, CRB113, and CRB113Wzc_Y572D_. (**A**) Survival rate of mice. BALB/c mice were intraperitoneally inoculated with 2 × 10^7^ CFU. *n* = 10/group for these groups; *n* = 3/group for control groups. Positive control group: hvKP American Type Culture Collection 43816; negative control group: phosphate-buffered saline (PBS). The significance was calculated using the log-rank test. *P < 0.05 and ****P < 0.0001. (**B**) Histological images of hematoxylin and eosin (H&E) stained liver tissue sections. BALB/c mice were intraperitoneally inoculated with 2 × 10^7^ CFU. H&E-stained liver tissue sections from mice at 7 h after infection. *n* = 3/groups. Negative control group: PBS. (**C**) Profiling of levels of cytokines. BALB/c mice were intraperitoneally inoculated with 2 × 10^7^ CFU. Serum from mice at 7 h after infection. Negative control group: PBS. *n* = 3/groups; bars indicate mean ± SD. ns *P* ≥ 0.05, **P < 0.01 and ****P < 0.0001. (**D**) Phagocytosis by RAW264.7 macrophages. *n* = 3 biological replicates; bars indicate mean ± SD. ****P < 0.0001. (**E**) Survival in normal human serum for 1 and 3 h. *n* = 3 biological replicates; bars indicate mean ± SD. (**F**) Growth curve. *n* = 3 biological replicates; bars indicate mean ± SD.

As CPS critically influences bacterial virulence by modulating phagocytosis and complement-mediated bactericidal activity, we assessed the virulence of three strains. Mice infected with the HMV strains CRB116 and CRB113Wzc_Y572D_ showed higher lethality, along with more pronounced inflammatory cell infiltration and more severe tissue damage, compared with mice infected with the NMV strain CRB113 (Fig. 3). Serum analysis of infected mice showed that the HMV strains CRB116 and CRB113Wzc_Y572D_ induced significantly higher cytokine release (Fig. 3). *In vitro* assays revealed that the HMV strains CRB116 and CRB113Wzc_Y572D_ were less susceptible to phagocytosis than the NMV strain CRB113 (Fig. 3). Unexpectedly, the serum resistance assay revealed that these HMV strains displayed markedly attenuated serum resistance (Fig. 3). To determine whether Wzc mutation affected bacterial fitness, growth curves were compared among the three strains, and no significant differences were observed (Fig. 3).

### Impaired serum resistance enhances LPS release

**Fig 4:**
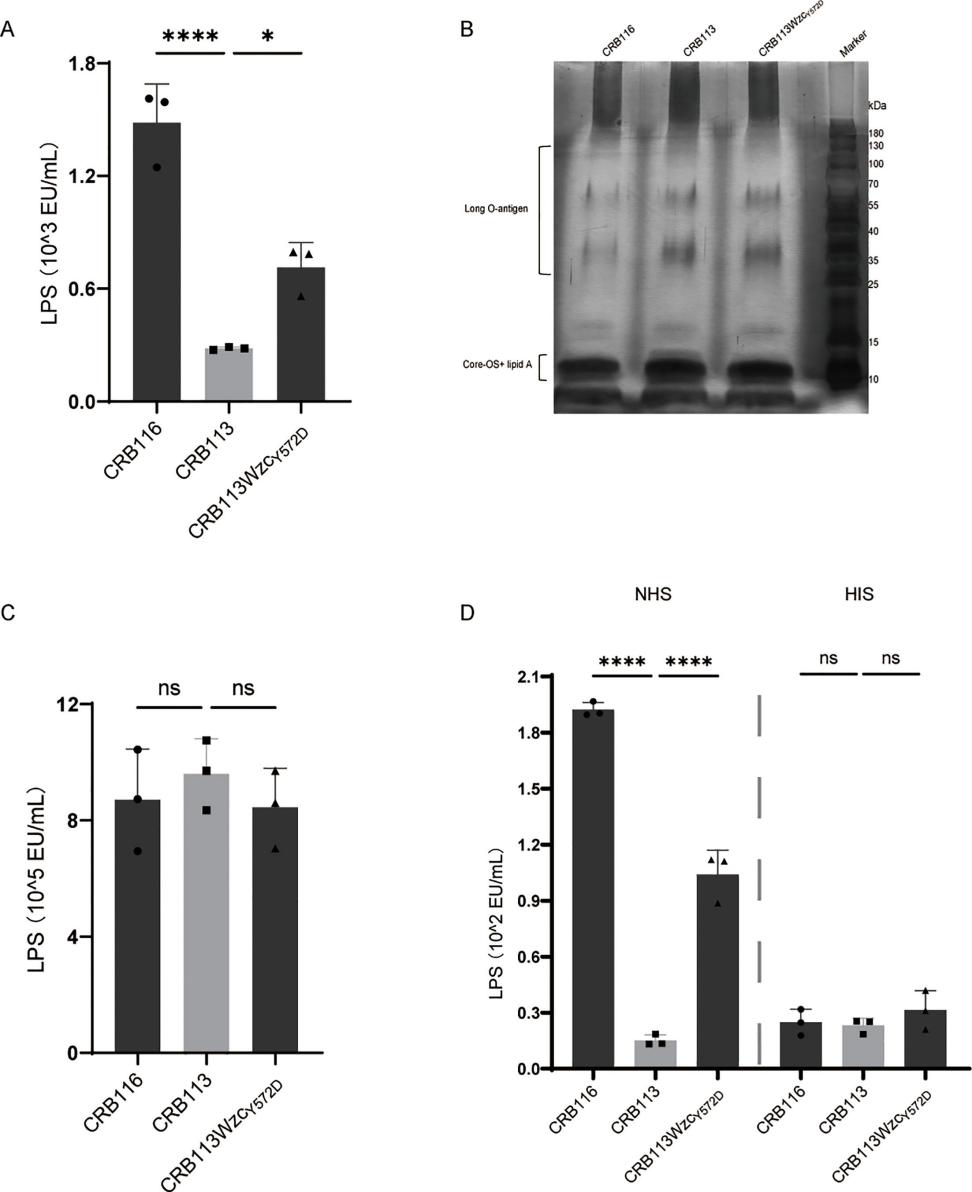
Lipopolysaccharide (LPS) analysis of CRB116, CRB113, and CRB113Wzc_Y572D_. (**A**) Quantification of LPS levels from infected mice serum at 7 h after infection. BALB/c mice were intraperitoneally inoculated with 2 × 10^7^ CFU. *n* = 3/groups; bars indicate mean ± SD. *P < 0.05 and ****P < 0.0001. (**B**) LPS silver staining. (**C**) Quantification of LPS production in bacteria. *n* = 3 biological replicates; bars indicate mean ± SD. ns *P* ≥ 0.05. (**D**) Quantification of serum-induced LPS release from bacteria. *n* = 3 biological replicates; bars indicate mean ± SD. ns *P* ≥ 0.05, ****P < 0.0001.

LPS contributes to bacterial fitness and pathogenesis. We quantified LPS levels in serum from mice infected with the three strains using the Limulus Amebocyte Lysate (LAL) test. Mice infected with the HMV strains CRB116 and CRB113Wzc_Y572D_ exhibited significantly higher serum LPS levels than those infected with the NMV strain CRB113 (Fig. 4).

To determine whether this increase resulted from altered LPS synthesis, we analyzed LPS structures and production. Structural analysis revealed that all three strains possessed intact O-antigen, core oligosaccharide, and lipid A components (Fig. 4). Quantification of bacterial LPS production revealed no significant differences among the three strains (Fig. 4). We next incubated the strains with serum and measured the LPS levels in the supernatant. Elevated LPS levels were detected in normal human serum (NHS) after incubation with HMV strains CRB116 and CRB113Wzc_Y572D_ (Fig. 4). In contrast, no significant differences were observed in heat-inactivated serum (HIS) following incubation with any of the strains (Fig. 4). Together, these results demonstrate that the Wzc_Y572D_ mutation does not affect LPS production but potentiates complement-mediated bacterial lysis, leading to the LPS release.

### C5b-9 deposition promotes LPS release

**Fig 5:**
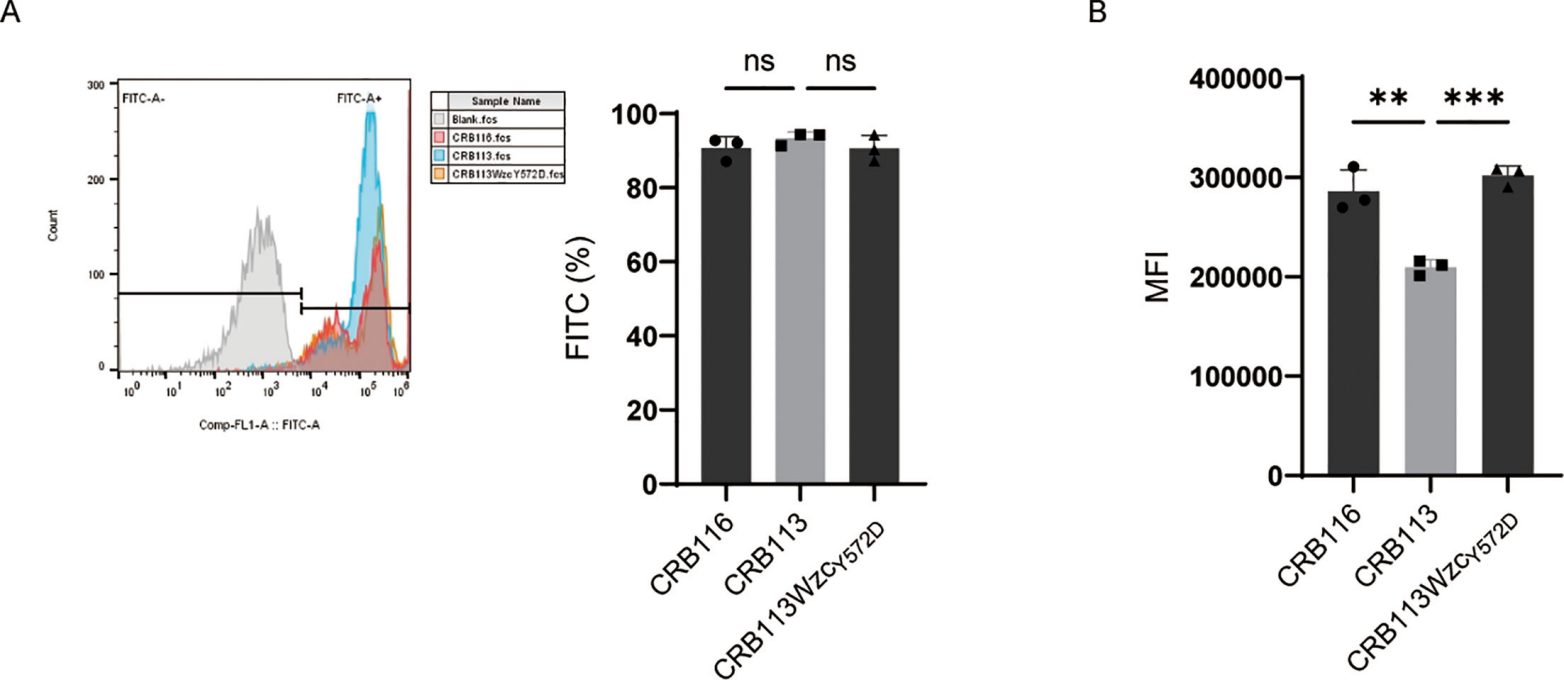
C5b-9 deposition of CRB116, CRB113, and CRB113Wzc_Y572D_. (**A**) Frequency of FITC-positive cells. *n* = 3 biological replicates, bars indicate mean ± SD. ns *P* ≥ 0.05. (**B**) Mean fluorescence intensity (MFI) of C5b-9 deposition on bacterial cells. *n* = 3 biological replicates, bars indicate mean ± SD. **P < 0. 01 and ***P < 0.001.

Activation of the complement system can directly kill bacteria through assembly of the membrane attack complex (MAC) on the outer membrane (23). We therefore analyzed C5b-9 deposition on the bacterial surface. C5b-9 deposition was detected on all tested strains, but deposition levels differed significantly (Fig. 5). The HMV strains CRB116 and CRB113Wzc_Y572D_ exhibited significantly stronger C5b-9 binding than the NMV strain CRB113 (Fig. 5). Increased MAC deposition is expected to enhance complement-mediated bacterial lysis, thereby promoting LPS release, consistent with our previous observations.

### Inhibition of TLR4 signaling by TAK-242 reduces LPS-mediated lethality

**Fig 6:**
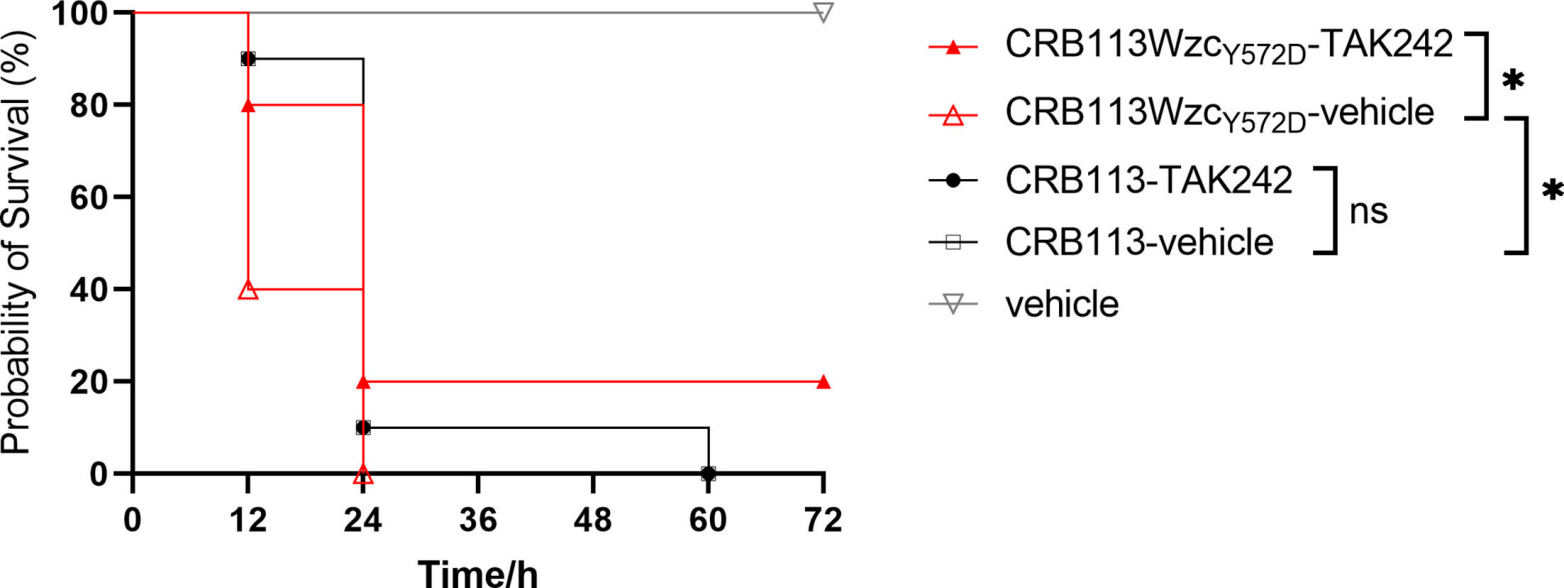
Survival rate of mice. BALB/c mice were intraperitoneally inoculated with 8 × 10^7^ CFU. *n* = 10/group for these groups; *n* = 3/group for vehicle group. Mice were pretreated with TAK-242 (3 mg/kg) or vehicle (phosphate-buffered saline containing DMSO) by intraperitoneal injection 1 h before bacterial challenge. The significance was calculated using the log-rank test. ns *P* ≥ 0.05, *P < 0.05.

Toll-like receptor 4 (TLR4), the primary receptor for bacterial LPS, is a central mediator of host immune response to infection. TAK-242 is a specific inhibitor of TLR4 signaling known to prevent LPS-induced systemic inflammation in mice (24). To investigate the role of LPS in virulence, mice were treated with vehicle, bacterial strain plus vehicle, or bacterial strain plus TAK-242. Mice infected with the HMV strain CRB113Wzc_Y572D_-vehicle showed higher lethality than those infected with the NMV strain CRB113-vehicle, consistent with our previous observations (Fig. 6). Pretreatment with TAK-242 significantly reduced lethality in mice infected with the HMV strain CRB113Wzc_Y572D_ (Fig. 6). In contrast, TAK-242 pretreatment had no significant effect on lethality in mice infected with the NMV strain CRB113 (Fig. 6). Together, these results demonstrate that inhibition of TLR4 signaling reduces LPS-mediated lethality.

## DISCUSSION

Distinct mucoid traits confer divergent survival strategies in pathogenic bacteria. Consistent with previous reports, our findings demonstrate that Wzc mutations confer an HMV phenotype and enhance virulence in CRKP. However, the functional consequences of Wzc-mediated HMV phenotype in CRKP have remained unclear. Here, we show that this phenotypic adaptation impairs serum resistance, leading to complement-mediated bacterial lysis and subsequent LPS release. This strategy ultimately potentiates virulence. These results provide novel insights into the regulatory relationship among CPS biosynthesis, the HMV phenotype, and virulence in CRKP ST11-KL64.

Our data identify Wzc mutations as a key determinant of HMV phenotype in CRKP ST11-KL64 independent of the *rmp* locus. In this genetic background, *rmpA/A2* appears to play a limited role in regulating mucoviscosity in CRKP, consistent with previous observations that its effect is strain-dependent (11, 20, 25). Mechanistically, the Wzc mutation promoted the HMV phenotype by altering CPS spatial distribution and chain length. The observed cell-free CPS release and uniform CPS length may be associated with Wzc mutations in the functional region, potentially through impaired cycling of Wzc between phosphorylated monomer and dephosphorylated octamer or disrupted interactions with Wza, Wzb, or Wzy (13, 18). Similar CPS alterations associated with Wzc mutations have been reported across multiple serotypes, suggesting convergent phenotypic outcomes in phylogenetically distinct strains (11, 12, 16).

Escape from phagocytosis and resistance to complement-mediated killing are two major virulence strategies in *Klebsiella pneumoniae*. In our study, the Wzc mutation-mediated HMV phenotype significantly reduced macrophage phagocytosis, likely due to the altered capsular architecture shielding bacterial surface epitopes, thereby promoting dissemination and survival *in vivo* (19, 26). This mechanism has been widely associated with increased virulence in HMV strains (19, 20).

Paradoxically, this phenotypic adaptation impaired serum resistance. In gram-negative bacteria, bacterial lysis results in LPS release. LPS consists of three main structural parts: lipid A, oligosaccharide core, and O-antigen. Critically, the lipid A is endotoxin and the main virulence factor (27). As a potent activator of the innate immune system, lipid A triggers a complex cascade of signaling pathways and inflammatory responses. TLR4, the cellular ligand for lipid A, plays a central role in the response to infection. TLR4-mediated signaling results in activation of the transcription factor nuclear factor-κB and production of proinflammatory cytokines such as TNF-α and IL- 6 (24). However, this excessive activation causes cellular death and tissue damage (28). We therefore investigated whether reduced serum resistance in Wzc-mediated HMV strains promoted bacterial lysis and LPS release, leading to increased virulence. Indeed, mice infected with these strains exhibited significantly higher serum LPS levels, which were not attributable to increased bacterial LPS synthesis but rather to complement-mediated bacterial lysis. Consistently, mice infection with the Wzc-mediated HMV strains resulted in increased cytokine production, more extensive inflammatory infiltration, and severe tissue damage. Additionally, the inhibition of TLR4 signaling reduced LPS-induced lethality in mice. Collectively, these findings indicate that the Wzc-mediated HMV strains impaired serum resistance paradoxically amplifies virulence by promoting LPS-driven inflammatory pathology. Therefore, we proffered a perspective that these adaptive strategies equip bacteria with a dual-functional arsenal: (i) a strong defense: Wzc-mediated HMV phenotype confers phagocytosis resistance to survive, and (ii) a good offense: Wzc-mediated HMV phenotype potentiates complement-mediated lysis of bacteria LPS release to cause tissue damage. This synergistic integration collectively increases bacterial virulence.

The pronounced effect of Wzc-mediated HMV phenotype on complement-mediated bacterial lysis raises important questions regarding its interaction with the complement cascade. Upon complement activation, the central component C3 is cleaved, and the activated fragment C3b covalently binds to the target surface to form the C5 convertase. Cleavage of C5 generates C5b, which will bind C6 to form the C5b6 complex, which associates sequentially with C6, C7, C8, and multiple copies of C9 to assemble the MAC (23). The O-antigen chain length is known to serum resistance, with strains expressing rough LPS (low molecular weight) being more susceptible to serum killing than those expressing smooth LPS (high molecular weight) (29). However, our structural analysis of LPS revealed no significant differences among the three strains. In contrast, Wzc-mediated HMV strains exhibited marked alterations in CPS spatial distribution and chain length. These CPS rearrangements may expose antibody-accessible epitopes on the bacterial surface, thereby facilitating C3 activation, MAC deposition, and/or MAC insertion into the outer membrane (23). Additionally, alterations in CPS composition may further promote C3 activation and/or functional MAC assembly (23, 30, 31). Consistent with this notion, strains expressing mannobiose- or rhamnobiose-containing CPS repeating units have been reported to exhibit reduced serum resistance (30). Nevertheless, the precise molecular mechanisms linking CPS architecture to complement susceptibility remain to be elucidated.

Overall, Wzc mutations in CRKP ST11-K64 remodel CPS spatial distribution and chain length, driving acquisition of the HMV phenotype and enhancing virulence through multiple, interconnected mechanisms. Clinical risk assessment of wzc variants should integrate patient outcomes with comprehensive epidemiological surveillance to better predict pathogenic potential.

## MATERIALS AND METHODS

### Clinical isolates collection and culture conditions

This study was approved by the Ethics Committee of Chinese PLA General Hospital (No. S2024-777-01). A total of 299 non-duplicate CRKP strains causing BSI were collected in a tertiary hospital (The First Medical Center of Chinese PLA General Hospital) in China from January 2018 to February 2023. Only the first strain isolated from patient was collected. Initial species identification was performed by VITEK MALDI-TOF MS (bioMérieux). The hvKP reference strains American Type Culture Collection (ATCC) 43816 (K2) were used in selected experiments. All bacterial isolates were cultured on Columbia blood agar or Luria-Bertani (LB) agar at 37°C; when grown in LB broth, they were incubated at 200 rpm and 37°C. Strains harboring thermosensitive replicon plasmids were exclusively maintained at 30°C to ensure plasmid stability. When appropriate, antibiotics were added at the following concentrations: apramycin (50 µg/mL) and hygromycin B (100 µg/mL).

### WGS analysis and phylogenetic analysis

All isolates were subjected to whole-genome sequencing (WGS) and assembly as described previously (32). The software Kleborate and Kaptive were used to identify the species, STs, KLs, virulent genes (*ybt*, *iro*, *iuc*, *rmp*, and *rmpA2*) (33, 34). Phylogenetic analysis was performed on all ST11-KL64 isolates as described previously (32). SNPs in *wzc* (KL64 reference strain genome sequencing is obtained from Kaptive software database. RefSeq: AB924600) were analyzed with snippy (https://github.com/tseemann/snippy).

### Sedimentation assay

Mucoviscosity was quantified using a sedimentation assay as previously described with minor modifications (7). Briefly, the optical density at 600 nm (OD_600_) of bacterial overnight cultures was quantified pre-centrifugation and post-centrifugation (1 mL) at 1,000 × *g* for 5 min. Sedimentation values were recorded as the post-/pre-centrifugation OD_600_ ratio. Isolates with a sedimentation value of 0.2 or greater are considered HMV phenotype, while those with values lower than 0.2 are NMV phenotype.

### Gene mutation construction

Mutation construction was performed using the CRISPR/Cas9 editing method, as previously described (20, 35). To construct *wzc*_T1714G_ in CRB113, the pSGKP-Hyg-*wzc*-spacer-donor plasmid was constructed via cloning 20-bp spacer sequence and amplifying wild-type *wzc* as the donor DNA for homologous recombination fragments from CRB116 into pSGKP-Hyg. The resulting pSGKP-Hyg-*wzc*-spacer-donor plasmid was electroporated into the pCasKP-harboring CRB113. Mutants were confirmed via PCR and Sanger sequencing. Plasmids and primers used are listed in Table S3.

### Extraction and quantification of CPS

Uronic acid was quantified to assess CPS production (10, 12, 19). For extracting cell-total CPS, 500 µL of the overnight culture was mixed with 100 μL of 1% Zwittergent 3-14 detergent in 100 mM citric acid (pH 2.0) and heated at 50°C for 30 min. Cell-free CPS was extracted by mixing 500 µL of the overnight culture with 100 µL of ultra-pure water instead of Zwittergent 3-14 detergent. The samples were centrifuged for 5 min at 17,000 × *g*, and 250 μL of the supernatant was transferred to a new tube and precipitated with 1 mL ethanol at 4°C for 30 min. The precipitate was retained and resuspended in 100 μL of ultra-pure water, and 600 μL 12.5 mM tetraborate-sulfuric acid was then added. The mixture was boiled for 10 min, and the OD_520_ was measured using 10 μL 0.15% phenylphenol. Uronic acid was determined from a standard curve generated with glucuronolactone.

### CPS chain length visualization

SDS-PAGE was performed to visualize CPS chain length as previously described (10, 12). The extraction of cell-total CPS and cell-free CPS was mentioned above. For cell-associated CPS, 500 µL of the overnight culture was collected and centrifuged at 21,000 × *g* for 15 min to wash the cells. The pellets were resuspended in 500 µL phosphate-buffered saline (PBS) and mixed with 100 μL of 1% Zwittergent 3-14 detergent in 100 mM citric acid (pH 2.0) and heated at 50°C for 30 min. Following centrifugation, 250 µL of the supernatant was collected and subjected to ethanol precipitation. The resulting pellet was resuspended in 100 µL of 1× SDS-PAGE loading buffer and heated at 100°C for 10 min. Subsequently, the samples were digested with proteinase K at 55°C overnight. Electrophoresis was carried out on 6% acrylamide resolving gels using Tris-glycine running buffer under constant voltage (300 V) for 4.5 h at 4°C. After electrophoresis, the gel was rinsed thoroughly with ultrapure water and stained with alcian blue solution (0.25% dye in 2% acetic acid) for 1 h. Excess stain was removed by destaining in a fixative solution (30% ethanol, 10% acetic acid) overnight. Finally, the gel was subjected to silver staining using the Pierce silver stain kit according to the manufacturer’s instructions.

### Transmission electron microscopy

Bacterial CPS was examined by transmission electron microscopy as previously described (19). Briefly, bacterial colonies grown overnight on Columbia blood agar plates were harvested and fixed in 2.5% glutaraldehyde at 4°C overnight prior to processing for imaging.

### Growth curve assay

Growth curves were established to compare the different bacterial fitness costs (22). The overnight cultures were diluted to 1.0 × 10^5^ CFU/mL in fresh LB broth, and then the absorbance was measured at OD_600_.

### Virulence evaluation in mouse model

All animal experiments in this study were approved by the Animal Ethics Review Committee of Novel Science (Beijing) (project number NWAKLL-2024-10-015). The mouse peritonitis-septicemia model was established with 6- to 8-week-old female BALB/c mice to assess the virulence of different strains (22). In the survival curve test, 10 mice were included in each test group and 3 mice in each control group (positive control group: hvKP ATCC 43816; negative control group: PBS). The mice were intraperitoneally infected at a dose of 2 × 10^7^ CFU. All mice were monitored every 12 h for 3 days to record survival rate. The livers and blood were collected at 7 h after infection. Livers were processed into histological slide for hematoxylin and eosin (H&E) staining. Blood samples were centrifuged to collect serum for cytokine and LPS quantification. For inhibition of TLR4 signaling, mice were pretreated with TAK-242 (3 mg/kg) or vehicle (PBS containing DMSO) by intraperitoneal injection 1 h before bacterial injection (24). LPS-driven hyperinflammation via the TLR4 pathway is a core mechanism in early sepsis mortality (36). We conducted dose-ranging studies to establish an acute sepsis model and determined 8 × 10^7^ CFU as the optimal infectious dose.

### Macrophage phagocytosis assay

A phagocytosis assay was performed to evaluate the ability of strains to inhibit phagocytosis by immune cells (20, 37). RAW264.7 macrophages were seeded in a 12-well plate at 5 × 10^5^ cells per well and incubated overnight in Dulbecco’s Modified Eagle Medium supplemented with 10% heat-inactivated fetal bovine serum at 37°C and 5% CO_2_. The overnight cell medium was refreshed using fresh medium and inoculated with bacteria for 1 h at a multiplicity of infection of 50. After washing with PBS, the cells were incubated in fresh medium containing 100 mg/L hygromycin for 1 h to clear extracellular bacteria. The cells were lysed with 500 µL 0.2% Triton X-100 for 10 min, and 100 µL of the lysate was plated for bacterial CFU determination.

### Serum resistance assay

The serum resistance ability of the strains was evaluated as previously described (38). Bacterial cells (2 × 10^6^ CFU/mL) were incubated with 75% NHS at 37°C for 1 and 3 h. Bacterial counts were determined to enumerate the survival rate. The survival rate was calculated using the following formula: survival rate = (CFU surviving at post-complement treatment/CFU surviving at initial) × 100.

### Extraction and visualization of LPS

LPS was extracted using two complementary extraction methods as previously described (39). Briefly, bacterial suspensions with an OD_600_ of 0.4 were centrifuged to collect the cells. Cell pellets were resuspended in 100 µL of lysis buffer. Subsequently, the cells were lysed at 100°C for 10 min, and protein digestion was performed. Samples were collected for SDS-PAGE analysis and LPS quantification. Samples were separated via SDS-PAGE at 150 V for 45 min and visualized by silver staining.

### LPS quantification

The bacteria LPS was quantified using the LAL test. To determine LPS released through complement-mediated bacterial lysis, bacterial cells (2 × 10^8^ CFU/mL) were incubated with 75% NHS and HIS at 37°C for 30 min. Subsequently, the mixture was centrifuged, and the supernatant was collected for LPS quantification. HIS was generated by incubating NHS for 30 min at 56°C.

### Detection of C5b-9 deposition on bacterial surfaces

The deposition of C5b-9 on the strains was assessed using flow cytometry (29). Bacterial cells (2 × 10^9^ CFU/mL) were incubated with 75% NHS for 30 min at 37°C and then washed with PBS. The cells were incubated with the anti-human C5b-9 antibody for 45 min, followed by washing and detection with a secondary antibody (FITC).

### Data analysis

All statistical analyses were conducted using GraphPad Prism software (version 8.0). For comparisons among multiple groups, one-way analysis of variance was applied to calculate *P*-values. Survival curves were generated using the Kaplan-Meier method, and differences between groups were assessed with the log-rank test. The thresholds for statistical significance were defined as follows: not significant (ns) *P* ≥ 0.05, *P < 0.05, **P < 0.01, ***P < 0.001, and ****P < 0.0001.

## SUPPLEMENTAL MATERIAL

Supplemental material spectrum.02847-25-s0001.xlsx Tables S1 to S3.

ASM does not own the copyrights to Supplemental Material that may be linked to, or accessed through, an article. The authors have granted ASM a non-exclusive, world-wide license to publish the Supplemental Material files. Please contact the corresponding author directly for reuse.
